# Betel Nut Usage Is a Major Risk Factor for Coronary Artery Disease

**DOI:** 10.5539/gjhs.v6n2p189

**Published:** 2013-12-27

**Authors:** Muhammad Shahzeb Khan, Faizan Imran Bawany, Muhammad Umer Ahmed, Mehwish Hussain, Asadullah Khan, Muhammad Nawaz Lashari

**Affiliations:** 1 Dow Medical College, Pakistan; 2 Ziauddin Medical University, Pakistan; 3 Civil Hospital, Pakistan

**Keywords:** betel nut, coronary artery disease, risk factor, Karachi, Pakistan

## Abstract

**Aim:**

The objective of our study was to assess betel nut usage as one of the major risk factors associated with coronary artery disease.

**Methods:**

This case control study consisted of 300 controls and 300 cases. A structured questionnaire was administered to the participants to assess consumption of betel nut and confounding variables. A respondent was considered a regular consumer of betel nut if he/she consumed one or more pieces of betel nut every day for a period of greater than 6 months.

**Results:**

About 8 in 10 betel nut chewers developed coronary artery disease. After adjusting for diabetes and hypertension, the odds ratio analysis depicted 7.72 times greater likelihood for coronary artery disease in patients who chewed betel nut for more than 10 years.

**Conclusion:**

Our study concludes that betel nut chewing is a significant risk factor leading to the development of coronary artery disease.

## 1. Introduction

Betel nut chewing is classified as the fourth major source of addiction and abuse worldwide with betel nut chewers now contributing to greater than 10% of the total worldwide population (Boucher & Mannan, 2002; Gupta & Warnakulasuriya, 2002). It is approximated that aroundsix hundred million people chew betel nut worldwide (Pankaj, 2010). The practice of betel nut chewing is prevalent especially in Asian countries such as Pakistan, India, Bangladesh, Sri Lanka, and Indonesia and also in regions where betel chewing communities tend to migrate (Boucher & Mannan, 2002; Gupta & Warnakulasuriya, 2002).

Betel nut chewing has been found to be associated with an increased risk of hepatocellular carcinoma (Tsai, Jeng, & Chuang, 2004), oral (Zhang & Reichart, 2007) and esophageal cancer (Wu et al., 2006) and liver cirrhosis (Hsiao, Liao, Hsieh, & Wong, 2007). In addition, a strong association of betel nut chewing with obesity, hypertension, type 2 diabetes, hyperlipidemia, chronic kidney disease and metabolic syndrome has also been established (Chang et al., 2006; Yen et al., 2006; Tung et al., 2004; Kang et al., 2007). These findings therefore imply an increased propensity towards the development of coronary artery disease [CAD] with betel nut chewing.

Cardiovascular diseases contribute to more than 15 million deaths annually worldwide (WHO, 1999) with Pakistan alone contributing to 410/100000 of the mortalities due to CAD (Samad et al., 1992). Studies have established that people of the Indo Asian origin show the greatest susceptibility towards CAD (Gupta et al., 2006); therefore CAD is classified as the chief cause of mortality in the South Asian countries such as Pakistan. Despite the fact that these deaths could be avoided by simple measures such as healthy diet, avoiding tobacco and regular physical activity, the numbers are still seen to be rising and costing up to 200000 deaths from CAD in Pakistan annually (The News, 2012).

Furthermore, a strong involvement of inflammatory markers of cardiovascular disease such as matrix metalloproteinase 9 and C-reactive protein with betel nut chewing has also been highlighted in a few studies (Timms et al., 2002). Extensive literature review shows that few studies have focused on betel nut chewing as a significant factor causing CAD. Since both betel nut chewing and CAD are prevalent worldwide, we conducted a case control study to further establish an association of betel nut chewing as a major risk factor for CAD.

## 2. Methods

This case control study was approved by the Institutional Review Board and Ethical Committee of Dow University of Health Sciences. Informed written consent was taken from each participant and they were assured about confidentiality of their data. The sample population was collected over a period of six months from January 2013 to June 2013. CAD patients were selected from registry of Cardiology department, Civil Hospital Karachi. As a government hospital, it offers low cost cardiac care within reach of patients with diverse socioeconomic status. A sample of 300 cases of coronary heart disease and 300 controls was selected in order to establish a relationship, if any, between betel nut chewing and CAD. Control samples primarily consisted of individuals who were diagnosed negative for CAD by cardiologists in the same time span as the case patients. The controls did not have a past history of ischemic heart disease or coronary heart disease. It should be highlighted that a high proportion of peoplewith chest pain and shortness of breath visit cardiac centers to rule out cardiac disease either due to self-referral or referral by a general physician. Some controls were diagnosed for non-specific chest pain and gastric hyperacidity. A small number of patients were not given any diagnosis and symptoms of chest pain or shortness of breath were written in patient notes.

Sample was collected using the following inclusion criteria: age must be 40-75 years and must be a non-smoker and non-alcoholic. Smokers were said to be regular consumers if they smoked 10 cigarettes or more every day for a period of greater than 6 months. Smokers were excluded from the study as we wanted a relationship of specifically betel nut chewing and CAD. Similarly, drinkers were considered as regular consumers if they consumed alcohol more than once a week for greater than 6 months. Diagnosis for the patients was based on positive classical symptoms and positive diagnostic tests such as electrocardiogram, cardiac enzyme, exercise tolerance test and coronary artery angiogram. Exercise tolerance test was considered positive if there was a ST segment elevation or depression of > 1mm in 3 consecutive P-QRS-T complexes in 2 or >2 consecutive leads. Results of angiogram were analyzed by two experienced cardiologist. When there was conflict of diagnosis of CAD between the cardiologists, opinion of a third cardiologist was sought for final diagnosis.

A structured questionnaire was administered to the participants to assess consumption of betel nut and confounding variables. Participants were first presented a screening questionnaire to filter eligible cases and controls. This questionnaire covered basic demographic variables included age, gender, residence, smoking and alcohol status and heart disease status. Once eligibility of participants was confirmed, a questionnaire was administered to gather detailed information on betel nut usage, presence of other risk factors for coronary heart disease and socio-demographic information including marital status, education level, occupation and monthly earning as a substitution for economic status.

A respondent was considered a regular consumer of betel nut if he/she consumed one or more pieces of betel nut every day for a period of greater than 6 months. Further information was sought to determine when betel nut consumption started and the amount consumed every day. All types of betel nut consumption were included in the survey, including betel leaf (pan) or betel nuts alone. History of other conditions like hypertension, diabetes, stroke and CAD were also inquired. A consumer was considered a hypertensive if he/she had a history of hypertension or was currently taking antihypertensive drugs. A consumer was considered a diabetic if he/she had a history of diabetes, was on an oral sugar lowering agent or insulin therapy, or had a fasting glucose level> 126mg/dl.

IBM SPSS for Windows Software (SPSS, Chicago, IL, version 21) was used for statistical analyses. Characteristics and habits were presented in terms of frequencies and percentages. Chi-square test was performed to examine difference of proportions of demographic, clinical and betel nut’s consumption pattern between cases and controls. Uni-variable analysis was performed to obtain odds ratio for cases and controls for consumption pattern of betel nut. Multi-variable analysis was used to attain adjusted odds ratio using hypertension and diabetes as confounding factors for betel nut chewers.

## 3. Results

A total of 300 CAD cases were recruited in the study and equal numbers of healthy controls werealsotaken. Most of the cases (n = 273) and controls (n = 262) were married. Majority of participants in both groups had under primary (Cases: 48.4%, Controls: 51.6%) and secondary (Cases: 51.2%, Controls: 48.8%) education. Most of the cases and controls had low socio-economic status. The proportions of cases having low BMI (< 18.5 KG/m^2^) was 40.90%, normal BMI (18.5-27kg/m^2^) 50% and high BMI (>27kg/m^2^) 52%. Their control counterparts had similar proportion on these different BMI ranges (P = 0.41). The presence of CAD disease in family was almost equal in both the groups (P = 0.704). Patients with hypertension were more likely to have CAD (Case: n = 210, 78.4% v/s Control: n = 58, 21.6%: P < 0.0001). Similarly, development of CAD in diabetic patient was significantly more as compare to a non-diabetic (P < 0.0001) (Table 1).

**Table 1:** Comparison of demographic variables between coronary artery disease cases and healthy controls

		CAD Patients (n=300)	Healthy Controls (n=300)	*P* Value
**Age**	45-60	120 (50.60%)	117 (49.40%)	0.802
	>60	180 (49.60%)	183 (50.40%)
**Gender**	male	210 (51.5%)	198 (48.50%)	0.294
	female	90 (46.90%)	102 (53.10%)
**Marital Status**	married	273 (51.00%)	262 (49.00%)	0.14
	divorced	9 (56.30%)	7 (43.80%)
	widowed	18 (51.00%)	31 (49.00%)
**Body Mass Index**	<18.5	18 (40.90%)	26 (59.10%)	0.41
	18.5-27	180 (50.00%)	180 (50.00%)
	>27	102 (52.00%)	94 (48.00%)
**Education**	Under Primary	135 (48.40%)	144 (51.6%)	0.459
	secondary	144 (51.20%)	137 (48.8%)
	under graduate	15 (46.90%)	17 (53.10%)
	post graduate	6 (75.00%)	2 (25.00%)
**Socioeconomic Status**	low	180 (47.10%)	202 (52.90%)	0.164
	middle	99 (55.60%)	79 (44.40%)
	high	21 (52.50%)	19 (47.50%)
**Family History**	yes	75 (51.40%)	71 (49.60%)	0.704
	no	225 (49.60%)	229 (50.40%)
**Hypertension**	No	90 (27.10%)	242 (72.90%)	<0.0001
	yes	210 (78.40%)	58 (21.60%)
**Diabetes**	no	177 (42.90%)	236 (57.10%)	<0.0001
	yes	123 (65.80%)	64 (34.20%)

About 8 in 10 betel nut chewers developed CAD. The proportion of development of CAD was almost half among participants who did not chew betel nut. The crude odds ratio depicted 6.4 times greater chance of CAD if a person was a betel nut chewer. Participants who were chewing betel nut for more than 10 years, hadahigher chance of developing CAD (Crude OR: 8.16, 95% CI = 3.51–18.95). After adjusting for diabetes and hypertension, the odds ratio analysis depicted 7.72 times more likelihood of CAD in patients who had been chewing betel nut for more than 10 years. The patients who chewed more than 10 pieces a day were 4.31 times more likely to develop CAD. The adjusted odds ratio was not significantly different from the unadjusted ratio (Adj OR: 4.476, 95% CI: 1.46–13.76) (Table 2).

**Table 2:** Comparison of betel nut consumption between coronary artery disease cases and healthy controls

		Healthy Control	CAD Patients	*P* Value	Crude OR	95% CI		Adjusted OR*	95% CI	
**Betel nut**	No	266 (61.29%)	168 (38.71%)							
	Yes	34 (20.48%)	132 (79.52%)	<0.0001	6.401	4.192	9.774			
**Time of start**	less than 10 years	24 (44.40%)	30 (55.60%)							
	more than 10 years	10 (8.90%)	102 (91.10%)	<0.0001	8.16	3.514	18.95	7.718	2.357	21.86
**Amount**	less than 10 pieces a day	19 (38.80%)	30 (61.20%)							
	more than 10 pieces a day	15 (12.80%)	102 (87.20%)	<0.0001	4.307	1.955	9.489	4.476	1.455	13.775

* Adjusted for Hypertension and Diabetes.

## 4. Discussion

This is one of the few case control studies to illustrate the risk of betel nut chewing associated with CAD. After taking into account the known risk factors such as age, gender, BMI, family history of hypertension and diabetes, our study established that there was a significant risk of CAD associated with the habit of betel nut chewing amongst adults, specifically those who had been habitually chewing betel nut for more than 10 years and more than 10 pieces a day. Several other studies have also expressed this strong co-relation, however some of those studies were centered around men, in comparison to ours which illustrates the effects on both the genders (Lan et al., 2007; Lin et al., 2008; Yen et al., 2008; Guh, Chen, & Tsai, 2007).

Many studies have also shown a strong relationship between smokeless tobacco use with risk factors for cardiovascular diseases like hypertension and deranged lipid profiles. However, the results assessing the association between smokeless tobacco and cardiovascular diseases are not consistent (Boffeta et al., 2009; Rahman, Mahmood, & Spurrier, 2011). Many cohort studies (Bolinder et al., 1994; Henley et al., 2005; Yatsuya & Folsom, 2010) and some case control studies (Teo et al., 2006; Rahman & Zaman, 2008), have shown a strong positive association. In comparison, other cohort (Accortt et al., 2002; Hansson, Pederson, & Galanti, 2009; Janzon & Hedblad, 2009) and case control studies (Huhtasaari et al., 1992; Huhtasaari et al., 1999; Hergens et al., 2005) have not yet established such an association. Some studies carried out in the western part of the world have found a significant association (Bolinder et al., 1994; Henley et al., 2005) while others have not (Accortt et al., 2002; Hansson et al., 2009; Janzon & Hedblad, 2009). However, most of the studies in South Asian countries have highlighted the positive association. This could be attributed to the fact that South Asian smokeless tobacco products differ from western products in terms of constituents and are mostly used in conjunction with betel nut. Therefore, it can strongly be proposed that the reason for cardiovascular diseases in smokeless tobacco users could be attributed to betel nut. The strong impact of betel nut chewing in the subsequent development of CAD is highlighted by our research which found that almost 8 out of 10 betel nut chewers developed CAD.

According to a research carried out amongst older individual adults, people who had the habit of chewing betel nut were at a significantly increased risk of cerebrovascular disease and mortality hazard ratio (HR = 1.66, 95% CI: 1.19, 2.30) compared to those who did not (Lan et al., 2007). Another study established that there was a dose dependantrelationship between betel nut chewing and CAD (Tsai et al., 2012). This fact is consistent with our research which also showed that people who chewed more than 10 pieces of betel nut a day were 4.31 times more likely to get CAD in comparison to those who did not. Previously, chewing betel nut had been found to be the cause of many risk factors for CAD. One study established that chewing betel nut was associated with central obesity (Lin et al., 2009). However, our study did not find an association between betel nut chewing and obesity, keeping body Mass Index as a measure of obesity.

According to a research by Tseng et al. (2008), betel nut chewing was strongly linked with hypertension in patients with type 2 diabetes mellitus. Another study established a positive association between betel nut chewing and metabolic syndrome (Guh et al., 2006). These facts are consistent with our research which found diabetes and hypertension to be significantly associated with a risk of CAD in patients chewing betel nut. It can therefore be proposed that chewing betel nut may either be an independent factor associated with CAD or be a factor by influencing known risk factors for hypertension or diabetes.

There are many mechanisms which could help explain the missing links among betel nut chewing, atherosclerosis and CAD. Acreoline, the compound found in betel nut, has been reported to cause induction of COX-2 expression and increasing TIMP levels in studies done in vitro (Tsai et al., 2003). The compound hydroxychavicol was found to induce the production of reactive oxygen species and thereby mediating cell damage (Iverson et al., 1995). Another study established that the expression of IL-1 beta, IL8, IL 6 and tumor necrosis factor increased in human mononuclear cells after being treated with the extract of betel nut (Chang et al., 2009). Furthermore, the extracts of betel nut and quid have found to increase the cytotoxic effects on oxidized LDL towards aortic endothelial cells (Owen et al., 2007). These facts help propose that the reason for CAD in betel nut chewing individuals could be specifically attributed to atherosclerosis.

Although we have established that chewing betel nut was associated with a significant risk of CAD, our study has certain limitations. Firstly, the questionnaires lacked details about the type of preparation used and whether betel nut was chewed alongwith a combination of different compounds. Secondly, the study does not establish that as to which specific branch of the coronary artery was more affected in this habit. However, our study has highlighted a significant issue of betel nut chewing being a major risk factor for one of the most common diseases worldwide.

## 5. Conclusion

Our study concludes that betel nut chewing is a significant risk factor for the development of CAD. It was found that betel nut chewing increased the likelihood of CAD by almost 6times. Furthermore, it was also established that those who had been chewing betel nut for more than 10 years had an 8 fold increase in the risk of developing CAD. This shows that long term betel nut chewing should be considered a significantly important risk factor along with hypertension and diabetes for CAD. However, further clinical studies should be conducted to explore the exact pathogenesis of betel nut chewing causing CAD.
